# An immune deficient mouse model for mucopolysaccharidosis IIIA (Sanfilippo syndrome)

**DOI:** 10.1038/s41598-023-45178-0

**Published:** 2023-10-27

**Authors:** Kari Pollock, Sabrina Noritake, Denise M. Imai, Gabrielle Pastenkos, Marykate Olson, Whitney Cary, Sheng Yang, Fernando A. Fierro, Jeannine White, Justin Graham, Heather Dahlenburg, Karl Johe, Jan A. Nolta

**Affiliations:** 1 Stem Cell Program and Institute for Regenerative Cures, University of California Davis Health System, Sacramento, CA USA; 2 Comparative Pathology Laboratory, University of California Davis, School of Veterinary Medicine, Davis, CA USA; 3 ReMotor Therapeutics, Inc., San Diego, CA USA

**Keywords:** Stem cells, Diseases

## Abstract

Mucopolysaccharidosis III (MPSIII, Sanfilippo syndrome) is a devastating lysosomal storage disease that primarily affects the central nervous system. MPSIIIA is caused by loss-of-function mutations in the gene coding for sulfamidase (N-sulfoglucosamine sulfohydrolase/SGSH) resulting in SGSH enzyme deficiency, a buildup of heparin sulfate and subsequent neurodegeneration. There is currently no cure or disease modifying treatment for MPSIIIA. A mouse model for MPSIIIA was characterized in 1999 and later backcrossed onto the C57BL/6 background. In the present study, a novel immune deficient MPSIIIA mouse model (MPSIIIA-TKO) was created by backcrossing the immune competent, C57BL/6 MPSIIIA mouse to an immune deficient mouse model lacking Rag2, CD47 and Il2rg genes. The resulting mouse model has undetectable SGSH activity, exhibits histological changes consistent with MPSIIIA and lacks T cells, B cells and NK cells. This new mouse model has the potential to be extremely useful in testing human cellular therapies in an animal model as it retains the MPSIIIA disease phenotype while tolerating xenotransplantation.

## Introduction

Mucopolysaccharidosis type III (MPSIII), commonly referred to as Sanfilippo syndrome, is a lysosomal storage disease that primarily affects the central nervous system. MPSIII is caused by a deficiency in one of four enzymes required for the degradation of heparan sulfate. Four subtypes of MPSIII (A, B, C and D) are defined based on the enzyme affected^1^. MPSIIIA is caused by various mutations in the gene coding for sulfamidase (N-sulfoglucosamine sulfohydrolase/SGSH) and the subsequent SGSH enzyme deficiency. MPSIIIB is caused by a deficiency in α-N-acetylglucosaminidase (NAGLU), MPSIIIC by acetyl–CoA:α-glucosaminide N-acetyltransferase (HGSNAT) deficiency and MPSIIID by N-acetylglucosamine 6-sulfatase deficiency^1,2^. Disruption of the heparan sulfate degradation pathway, regardless of the enzyme responsible, leads to accumulation of heparan sulfate, neuroinflammation and the clinical symptoms of all types of MPSIII. While the clinical presentation is similar in all subtypes, studies suggest that MPSIIIA results in a more severe disease progression^1–3^. In infancy, heparan sulfate has not accumulated enough to result in a disease phenotype, thus infants appear healthy at birth and initially develop normally. Early symptoms of this disease become evident between 1 and 4 years of age and include developmental delays and behavioral issues. Children then suffer progressive dementia, hyperactivity, sleep disturbances, loss of verbal skills, loss of motor function and early death. The mean age of death for MPSIIIA patients is 15 years and there is currently no cure or disease modifying treatment option^1,4,5^.

Rodents are widely used to model human disease and test potential therapies due to their compact size, ease of handling, short reproductive cycle, and the ability to manipulate their genetics. In 1999 the first murine model for MPSIIIA was characterized by Bhaumik et al. on a mixed background that recapitulated many of the biochemical, pathological and clinical features of the disease seen in children^6^. It was later discovered that these mice carry a spontaneous missense mutation at the SGSH locus that gives rise to an amino acid change, reducing SGSH function by about 97%^7^. Further work by Lau et al. backcrossed the MPSIIIA mutation onto the C57BL/6 background to improve the stability of the line and ensure genetic homogeneity within the strain^8,9^.

Current treatment strategies for MPSIIIA focus on gene therapy and enzyme replacement therapy using a variety of approaches^10,11^. Intravenous enzyme replacement therapy has been proven to be an effective treatment option for other MPS diseases including MPSI, MPSII and MPSVI^1,12,13^. Since MPSIIIA is primarily a disease of the central nervous system, the blood–brain barrier poses a major obstacle in developing effective treatments. In order to bypass the blood–brain barrier, SGSH has been injected into the cerebral spinal fluid or directly into the brain of MPSIIIA mice with promising results^14,15^. However, these approaches would require repeat injection into the CSF or brain and would likely result in dramatic fluctuations in enzyme levels. Our group is interested in using human neural stem cells (NSC) for enzyme replacement therapy in MPSIIIA; therefore, we developed and characterized an immune deficient mouse model of MPSIIIA to allow testing of human cellular therapies in a relevant disease model.

## Results

### Generation of immune deficient MPSIIIA mouse model

**Figure 1:**
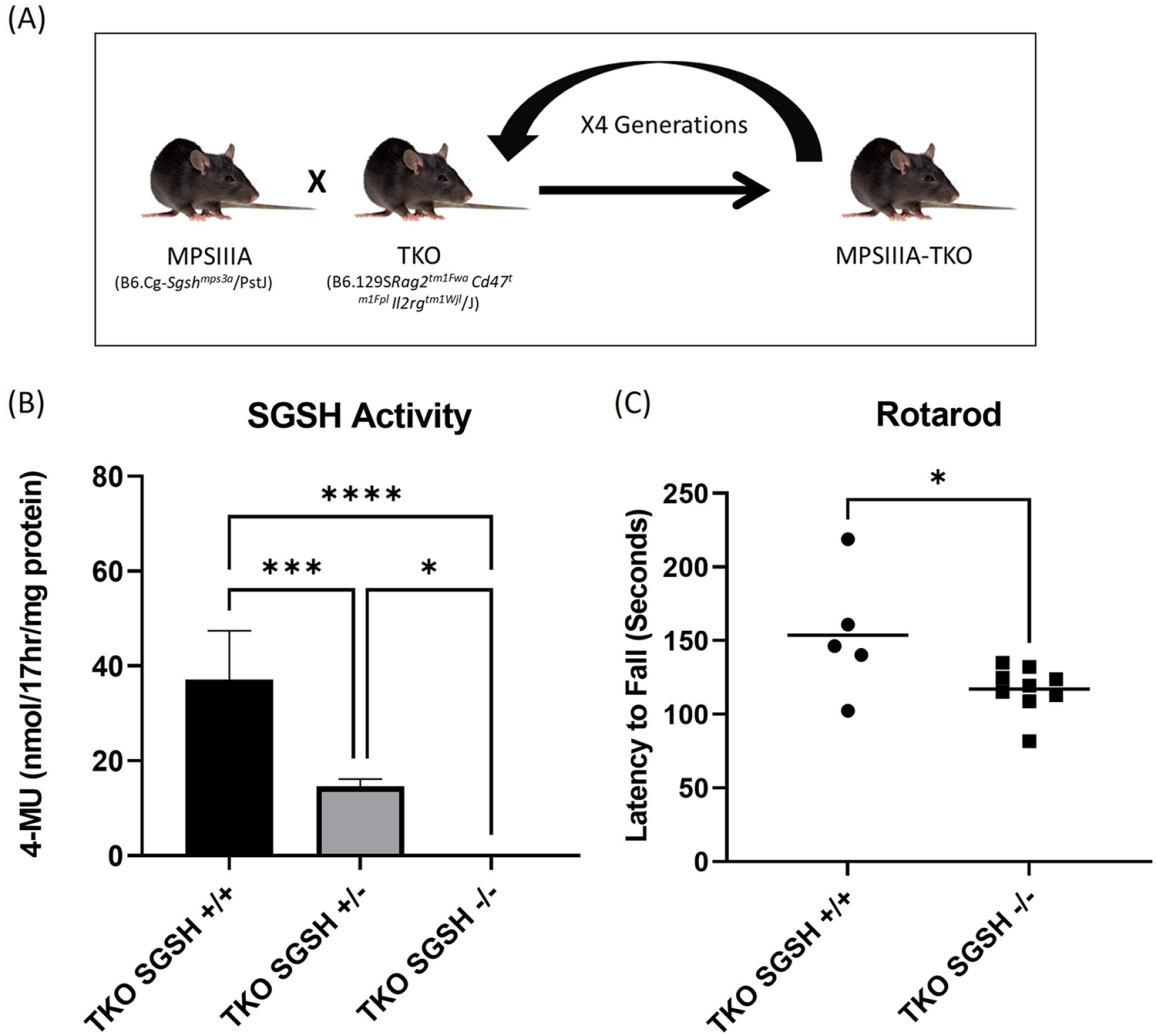
(**A**) The MPSIIIA-TKO mouse model was generated by backcrossing the immune competent MPSIIIA mouse model to an immune deficient model lacking Rag2, CD47 and Il2rg genes. After 4 generations, a breeding colony of SGSH+/−, Rag2−/−, CD47−/− and Il2rg−/− animals was established. (**B**) SGSH activity was measured in brain lysate from MPSIIIA-TKO mice at 5–6 months of age. SGSH activity was reduced in mice heterozygous for the SGSH mutation and was undetectable in mice homozygous for the SGSH mutation. One-way ANOVA revealed significant genotype differences F (2, 14) = 49.76, *p* < 0.0001. Post-hoc analysis revealed significant differences between groups as indicated *p = 0.0125, ***p = 0.0003 and ****p ≤ 0.0001 (n = 7 SGSH+/+, 4 SGSH+/−, 6 SGSH−/−). (**C**) Rotarod performance was evaluated at 10 weeks of age using the accelerating rotarod. Latency to fall was significantly reduced in the MPSIIIA-TKO SGSH−/− animals when compared to their MPSIIIA-TKO SGSH+/+ littermates (*p = 0.0349) (n = 9 SGSH+/+, 5 SGSH−/−).

A novel immune deficient MPSIIIA mouse model (MPSIIIA-TKO) was created by backcrossing the immune competent MPSIIIA mouse (Jax Stock No. 003780) to a triple knockout (TKO Jax Stock No. 025730) immune deficient mouse model lacking Rag2, CD47 and Il2rg genes. Animals were genotyped for Rag2, CD47, Il2rg and SGSH until a breeding colony of MPSIIIA-TKO (Rag2, CD47 and Il2rg), SGSH heterozygous breeders was established (Fig. 1A).

### MPSIIIA-TKO mice exhibit SGSH deficiency

An enzymatic SGSH assay was used to determine the SGSH activity levels in the brains of MPSIIIA-TKO animals. As expected, MPSIIIA-TKO SGSH−/− animals lack measurable SGSH activity, MPSIIIA-TKO SGSH+/+ littermates have normal SGSH activity levels and the heterozygous animals show diminished SGSH activity (Fig. 1B). Genotype had a significant impact on SGSH activity; we demonstrate a significant difference between MPSIIIA-TKO SGSH+/+ littermates and MPSIIIA-TKO SGSH+/− (*p* = 0.0003), significant difference between MPSIIIA-TKO SGSH+/+ littermates and MPSIIIA-TKO SGSH−/− (*p* ≤ 0.0001), and significant difference between MPSIIIA-TKO SGSH+/− and MPSIIIA-TKO SGSH−/− (*p* = 0.0125).

### MPSIIIA-TKO mice show motor deficits

To evaluate motor coordination in this novel strain, animals were tested on the accelerating rotarod. MPSIIIA-TKO SGSH−/− mice spent significantly less time on the rotarod than their MPSIIIA-TKO SGSH+/+ littermates (*p* = 0.0349) at 10 weeks of age (Fig. 1C).

### Histologic and hematologic changes in MPSIIIA-TKO mice support an immune deficient MPSIIIA phenotype

To further characterize this strain, 8 animals (4 MPSIIIA-TKO SGSH−/− and 4 MPSIIIA-TKO SGSH+/+ wildtype littermates) from the MPSIIIA-TKO colony were submitted to the UC Davis Comparative Pathology Laboratory at for complete blood counts, serum biochemistry analysis, necropsy and histopathology. The life expectancy of the parental MPSIIIA strain is 7–10 months; therefore, we evaluated mice at 8 months of age to capture the late-stage disease phenotype.

**Figure 2:**
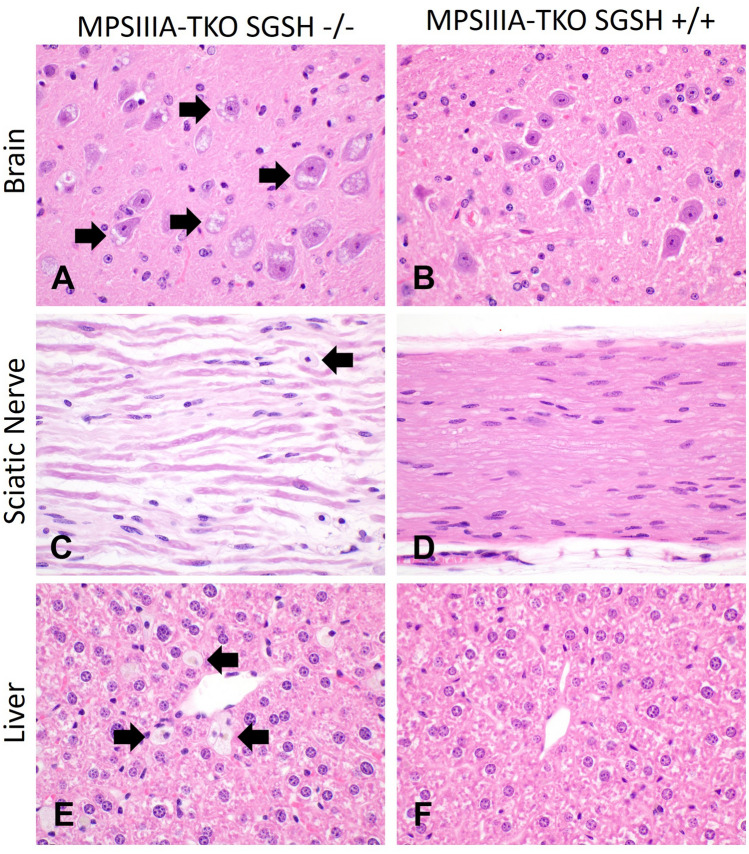
Cytoplasmic microvesiculation affecting epithelia, macrophages and neurons is consistent with a lysosomal storage disorder in 8-month-old MPSIIIA-TKO mice SGSH−/−. Fine clear vacuoles (black arrows) distend the cytoplasm of neurons (**A**), macrophages (**C**) and Kupffer cells (**E**). Additionally, there is increased space between axons in the sciatic nerve (**C**) that could correspond with dilation of myelin sheaths. Images of control tissues (**B**,**D**,**F**) from the MPSIIIA-TKO SGSH+/+ littermates are provided for comparison.

Significant histologic findings were similar among the four MPSIIIA-TKO SGSH−/− mice and consist of distention of the cytoplasm of numerous cell types by fine vesicles or vacuoles (Fig. 2). Affected cells include neurons, epithelia, and cells with a histiocytic or fibroblastic morphology. Cytoplasmic distention by vacuoles or vesicles is a hallmark of lysosomal storage disorders and is consistent with accumulation of a storage product. These findings are similar to those described for the immune competent strain by Bhaumik et al.^6^.

In the central nervous system, storage product is identified in neurons in the forebrain, midbrain, and hindbrain. Specifically, hindbrain neurons in the cerebral cortex (layers 5 and 6, somatomotor, somatosensory, and orbital areas), cortical subplate, cerebral nuclei (lateral septal nucleus, pallidum), thalamus, and hypothalamus (lateral zone, periventricular zone); midbrain neurons in motor related nuclei (midbrain reticular nucleus); hindbrain neurons in the cerebellum (fastigial nucleus, interposed nucleus), medulla (motor and sensory related nuclei).

In all four MPSIIIA-TKO SGSH−/− mice, storage product in presumptive histiocytic and fibroblastic cells is identified in the optic nerve, in association with axonal atrophy. In three MPSIIIA-TKO SGSH−/− mice, histiocytic and fibroblastic cells in the sclera contain storage product. In the ears of all four −/− mice, accumulated storage product is observed in histiocytic and fibroblastic cells in the otic ganglion.

Ganglion cells in the wall of the stomach and intestines are distended by storage product in three MPSIIIA-TKO SGSH−/− mice, and in histiocytic or fibroblastic cells in peripheral nerves in all four.

Cells with storage product are identified in the periosteum of bones of the skull, spinal column, sternum, and hind limbs of mutant mice. Affected cells are round or spindle-shaped, resembling either histiocytes or resident fibroblasts/mesenchymal cells. Cells distended by storage product are also identified in connective tissues supporting joints/bones, including the infrapatellar fat pad.

Cytoplasmic distention with microvesiculation or vacuolation was identified in all MPSIIIA-TKO SGSH−/− mice in epithelia of the following tissues: epithelium of the urinary bladder, ureters, endometrial glands, epididymides, seminal vesicles, prostate, clitoral gland, mammary gland, gallbladder, renal distal convoluted tubules and collecting ducts, pancreatic ducts, salivary gland ducts and acinar cells, parathyroid gland, and thyroid gland. In various tissues, this change was associated with individual or small, scattered clusters of apoptotic or necrotic cells. Other affected cell types include Kupffer cells, hepatocytes, vascular smooth muscle, neuronal bodies, leptomeninges, cartilage, and periosteal tissue (Table 1 and supplementary data).

**Table 1:** Grading reflects the relative number of cells with storage product in the tissue/organ; 1 = mild involvement, 2 = moderate involvement, 3 = severe involvement.

Tissue	Cell type(s) affected	Storage product	Distribution
Brain (forebrain, midbrain, hindbrain)	Neuronal, histiocytic/fibroblastic	1–2	Multifocal
Spinal cord	Neuronal, histiocytic/fibroblastic	3	Multifocal
Peripheral nervous system (hind limb)	Neuronal, histiocytic/fibroblastic	3	Multifocal
Enteric nervous system (stomach, large intestines)	Neuronal, histiocytic/fibroblastic	2	Multifocal
Ear (otic ganglion)	Histiocytic/fibroblastic	1	Multifocal
Eye (optic nerve, sclera)	Histiocytic/fibroblastic	2	Multifocal
Periosteum of skull, vertebrae, sternum, hind limb	Histiocytic/fibroblastic	2–3	Multifocal

Cytoplasmic vacuolation or microvesiculation in histiocytic or fibroblastic cells was identified in all MPSIIIA-TKO SGSH−/− mice in components of the following tissues, particularly the lamina propria, muscularis, and adventitia: urinary bladder, ureters, vagina, uterus, skin, testicular interstitium, penis, seminal vesicles, prostate, preputial/clitoral gland, mammary gland stroma, adipose tissue, ovarian interstitium, salpinx, adrenal cortex and capsule, skeletal muscle of the diaphragm, limbs, head, and tongue spinal nerves, peripheral nerves, gallbladder, spleen, renal interstitium, pancreatic interstitium, myocardium, gastrointestinal tract, white matter of the spinal cord, and optic nerve (Table 1 and supplementary data). The same change was identified in the larynx, sclera, tooth pulp cavity, and pituitary gland of one or more mutant mice. In addition to microvesiculation in nerves, marked separation of axons was observed. This change may represent a reduction in axonal diameter. Axonal swelling, degeneration, demyelination, and intramyelinic edema were not identified.

All MPSIIIA-TKO SGSH−/− mice were found to have severe bladder distension (up to 2 cm). While urinary bladder distention was also noted in both MPSIIIA-TKO SGSH+/+ male mice, changes consistent with accumulation of storage product were not identified. The cause of the distention in the MPSIIIA-TKO SGSH+/+ male mice could be due to urethral blockage with an agonal coagulum, a common finding in euthanized male mice.

Histologic changes consistent with an immunodeficient mouse strain were present in all mice submitted. Thymic lymphocyte depletion with cyst formation was observed in two of the animals; the absence of thymic tissue in the remaining mice was interpreted as lymphocyte depletion/atrophy. A shift towards increased erythroid and granulocytic precursors in bone marrow was also interpreted as consistent with an immunodeficient phenotype. All remaining histologic findings were interpreted as non-genotype associated and incidental.

**Figure 3:**
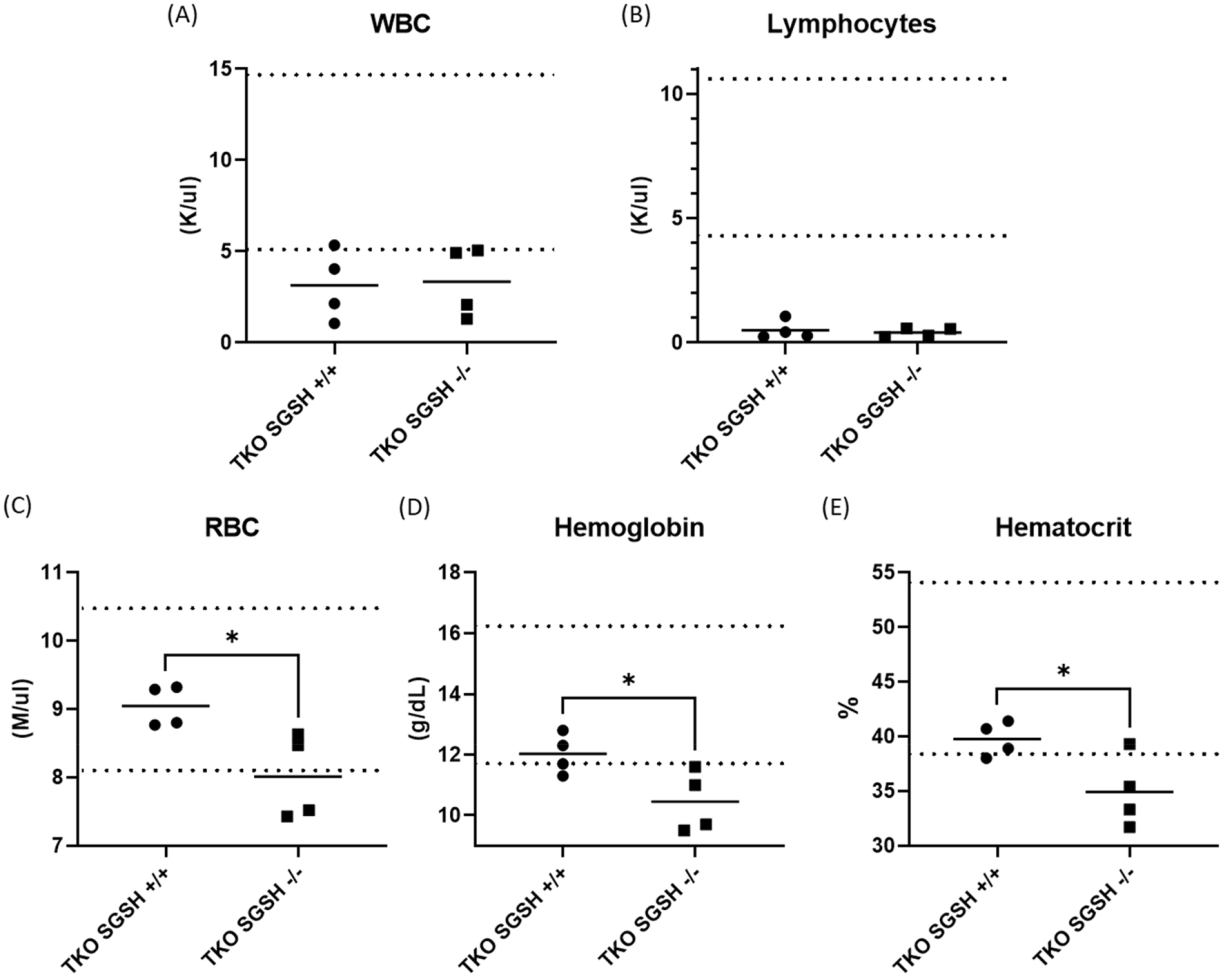
Hematological findings in 8-month-old MPSIIIA-TKO mice (n = 4/group). Dotted lines define the reference range. (**A**) White blood cell counts. (**B**) Lymphocyte counts. (**C**) Red blood cell counts. (**D**) Hemoglobin. (**E**) Hematocrit. *p < 0.05.

Hematological changes consistent with an immune deficient background were evident in all MPSIIIA-TKO mice, regardless of SGSH genotype. All mice submitted were deficient in white blood cells, specifically lymphocytes when compared to the reference ranges (Fig. 3). Hemoglobin, hematocrit and red blood cell counts were also significantly decreased in MPSIIIA-TKO SGSH−/− mice compared to their SGSH+/+ littermates (*p* = 0.0406, 0.0381 and 0.0247 respectively). Calcium levels were within the normal range for all mice submitted, but were significantly lower in MPSIIIA-TKO SGSH−/− mice as compared to their SGSH+/+ littermates (*p* = 0.0452, see supplemental data). All other lab values were non-genotype dependent, associated with immunodeficiency or incidental (see supplemental data).

### MPSIIIA-TKO mice lack key immune cells

**Figure 4:**
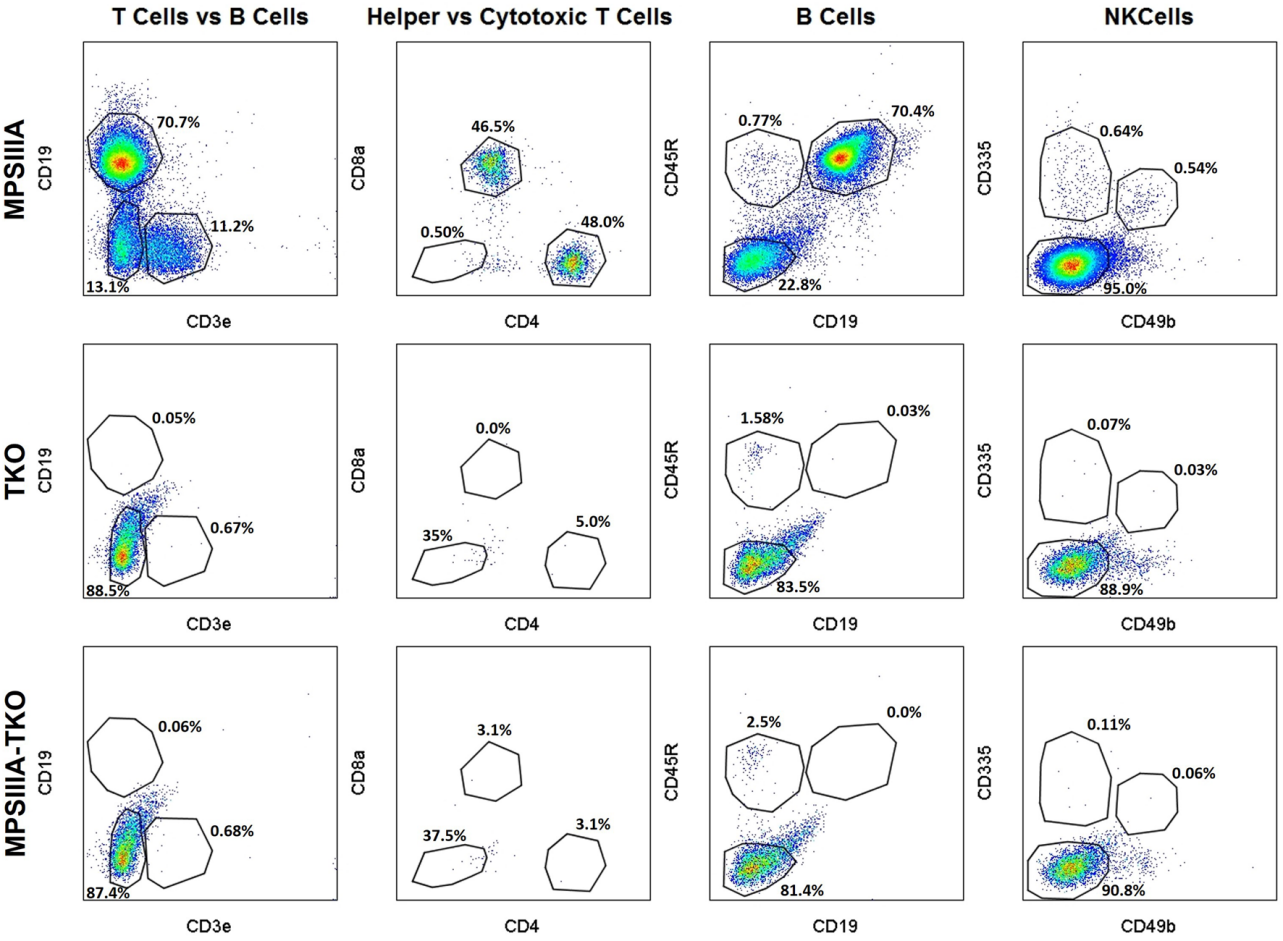
Flow cytometry was used to confirm the immune deficient status of the novel MPSIIIA-TKO strain. Splenocytes were isolated from 6-month-old immune competent MPSIIIA, immune deficient TKO and MPSIIIA-TKO SGSH−/− mice. The immune profile of our novel MPSIIIA-TKO strain matches that of the immune deficient TKO strain used to create this model. Columns 1, 3 and 4 include all CD45 + cells; Column 2 includes only T cells (CD3e+ CD19−).

A comprehensive flow panel was used to compare the immune profile of the novel strain to that of the parental strains. Cells isolated from the spleens were evaluated to assess the presence of T cells, B cells and NK cells. The immune profile of our novel MPSIIIA-TKO strain resembles that of the TKO parental strain in contrast to the immune competent MPSIIIA parental strain. The MPSIIIA-TKO mice lack helper T cells, cytotoxic T cells, B cells and NK cells as demonstrated in Fig. 4.

### Xenotolerance in MPSIIIA-TKO mice

**Figure 5:**
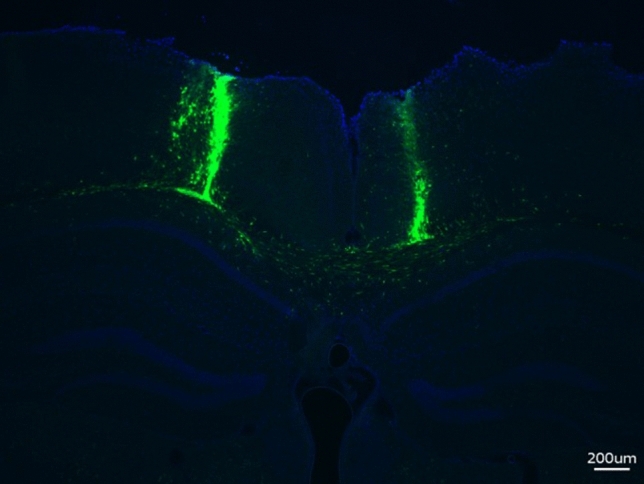
Human neural stem cell engraftment in MPSIIIA-TKO mice 8 weeks after transplantation. Cells are predominantly located in the white matter and needle tract; however, there is evidence of migration into the parenchyma. (Blue = DAPI, Green = GFP).

To evaluate the xenotolerance of this strain, human neural stem cells engineered to express green fluorescent protein (GFP) were bilaterally transplanted into the corpus callosum of MPSIIIA-TKO SGSH−/− mice. Eight weeks post transplantation, cell survival was robust. Cells appear to have traveled along the white matter tract and were predominately found within the corpus callosum and needle tract; however, there is evidence of migration into the parenchyma in several sections (Fig. 5). Cells were easily identified from 0.15 mm anterior to bregma to about 3.0 mm posterior to bregma (the most posterior section evaluated). This indicates that the cells were able to travel approximately 2 mm from the injection site (− 1.8 mm AP).

## Materials and methods

### Animals

Animals were group housed in a humidity/temperature-controlled pathogen-free barrier facility with a 12 h light–12 h dark cycle. Water and chow (Teklad global 18% protein 2918, Envigo) were provided ad libitum. All animal studies were approved by the UC Davis Institutional Animal Care and Use Committee and carried out in accordance with relevant guidelines and regulations. All methods are reported in accordance with ARRIVE guidelines. Animals were age-matched and assigned to groups based on genotype; therefore, randomization was not necessary. Groups were mixed sex and balanced to the best of our ability. Sample size was dictated by animal availability. Inclusion/exclusion criteria were not set and there were no exclusions from the presented analyses. The facility where this research was conducted is fully accredited by AAALAC, International.

### Strain development

An immune deficient MPSIIIA mouse model was created by backcrossing the immune competent MPSIIIA model (B6.Cg-Sgsh^mps3a^/PstJ Jax Stock No: 003780) to a triple knockout (TKO) immune deficient mouse model lacking Rag2, CD47, and Il2rg (B6.129S-*Rag2*^*tm1Fwa*^* Cd47*^*tm1Fpl*^* Il2rg*^*tm1Wjl*^/J JAX Stock No. 025730). Genotyping was performed for Rag2, CD47, Il2rg and SGSH by Transnetyx automated genotyping service using tail or ear clips. F1 animals heterozygous for the SGSH mutation were backcrossed to the TKO immune deficient strain until a breeding colony of TKO, SGSH heterozygous breeders was generated. This colony of MPSIIIA-TKO mice is maintained in a pathogen-free barrier facility using MPSIIIA-TKO SGSH+/− animals as breeders. Subsequent experiments were performed using MPSIIIA-TKO SGSH−/−, MPSIIIA-TKO SGSH+/−, and/or MPSIIIA-TKO SGSH+/+ animals.

### Flow cytometry

To determine the presence of immune cells in the MPSIIIA-TKO strain, spleens were collected and labeled with antibodies specific for mouse immune cell markers and analyzed by flow cytometry following a previously published protocol^16^. Mice were euthanized by CO_2_ asphyxiation. Spleens were harvested and placed onto a 70 μM strainer cap atop a 50 mL conical tube. Using a 3 mL syringe, each spleen was popped open and washed with 2 mL PBS. The spleens were repeatedly minced and washed with 2 mL PBS a total of 3 times. The cell suspension was transferred to a 15 mL conical and centrifuged at 2000 rpm for 4 min. The cell pellet was resuspended in 2.5 mL PBS and cell clumps were removed. Samples were aliquoted and stored on ice until ready to stain. All immune cells were labeled with BUV395-conjugated CD45 (clone 30F11) (BD Biosciences). T cells were labeled with an APC-Cy7-conjugated CD3e (clone 145-2C11), BUV737-conjugated CD4 antibody (clone RM4-5), and PECY7-conjugated CD8 antibody (clone 53-6.7) (BD Biosciences). B cells were labeled with an APC-conjugated CD45R antibody (clone RA36B2) and a BB515-conjugated CD19 antibody (clone 1D3) (BD Biosciences). NK cells were labeled with a PE-conjugated CD335 antibody (clone 29A1.4) and a BV421-conjugated CD49B antibody (clone DX5) (BD Biosciences). Compensation was completed using BD positive/negative compensation beads and an ArC Reactive Compensation bead kit. Viability was assessed with the ZOMBIE Aqua Fixable Viability Kit (BioLegend). Flow cytometry was performed on a BD Fortessa and collected data was analyzed using FloJo software (Beckman Coulter).

### SGSH enzyme activity

Mice were euthanized by CO_2_ asphyxiation followed by bilateral thoracotomy at 5–6 months of age. Mice were perfused with 10 mL PBS (HyClone) and brains were harvested, bisected, placed in 1.5 mL tubes, flash frozen in liquid nitrogen and stored at − 80 °C. Frozen brains (one hemisphere/animal) were homogenized with Omni Bead Ruptor 24 Elite using plastic beads (Omni International in 1 mL of RIPA lysis buffer (ThermoFisher) in 2 mL tubes. Protein concentration of each tube was determined by Micro BCA protein assay kit (ThermoFisher). 20 µg of protein from each homogenate was brought to 50 µL assay volume with water and SGSH activity was determined using 4MU-α-GlcNS substrate (Biosynth International) in Assay Buffer (Bis–Tris 28.6 mM/0.7% NaCL, pH 6.5), essentially as described by Whyte LS et al.^17^ and Karpova EA et al.^18^ with minor modifications. Briefly, the enzyme reaction was carried out in 8-well tube strips for PCR in 56µL total reaction volume for 17 h at 47 °C using a PCR machine (Eppendorf). The reaction was stopped by adding 11 µL of Stop Buffer (NaCitrate, 0.2 M /NaH2PO4 0.26 M/Na_2_HPO_4_ 0.14 M, pH 6.7). To release the 4MU moiety from the 4MU-α-GlcNH, 0.2 units of α-glucosidase from Bacillus stearothermophilus (Sigma-Aldrich) in 20 µL water was added to the reaction mixture and incubated for 24 h at 37 °C in the PCR machine. The whole volume of each tube (87 µL) was transferred to a well of 96-well plate and 100 µL of Read Buffer (Na_2_CO_3_ 0.5 M, pH 10.7) was added. The 4MU fluorescence was read at 360 nm excitation and 460 nm emission in SpectraMax Gemini XS fluorescent plate reader (Molecular Devices). Raw 4MU fluorescence values less than the background fluorescence were reported as zero. A standard curve of recombinant human SGSH (R&D Systems) and a serial dilution of free 4MU were included in each assay as positive controls.

### Histology and hematology

Animals were submitted to the UC Davis Comparative Pathology Laboratory for complete blood counts, serum biochemistry analysis, necropsy and histopathology of 50 tissues. A total of 8 immune deficient mice from the established MPSIIIA-TKO colony were submitted at 8 months of age: 4 SGSH−/− and 4 SGSH+/+ (2 male and 2 female each). Mice were euthanized by CO_2_ asphyxiation and cardiac exsanguination. Whole blood was collected for hematology and serum biochemistry. Automated complete blood counts were performed with a Drew HemaVet 950FS analyzer (Drew Scientific, Miami Lakes, Florida) following gentle mixing. Serum was prepared by centrifugation of whole blood at 3400 rpm for 10 min, then analyzed for alanine transaminase, aspartate transaminase, alkaline phosphatase, amylase, blood urea nitrogen, creatinine, glucose, total bilirubin, total protein, albumin, calcium, phosphorus, sodium, chloride, and potassium using a COBAS INTEGRA 400 plus analyzer (Roche, Basel, Switzerland). Hematologic and biochemical reference ranges provided were generated in the laboratory, from the analyzers, and using BALB/c mice.

A comprehensive set of tissues was immersion-fixed in 10% neutral-buffered formalin for 72 h. Bony tissues were decalcified by immersion in Formical 2000 (StatLab). Formalin-fixed decalcified tissues were processed routinely, embedded in paraffin, sectioned at 5 µM, and stained with hematoxylin and eosin. Tissue sections were evaluated by board-certified veterinary anatomic pathologists (Gabrielle Pastenkos, Denise Imai). All animals were submitted in a blinded manner and genotypes were only disclosed to the pathologists at the conclusion of the study in order to write an interpretation of the results.

### Rotarod

Mice were trained and tested on the rotarod as previously published^16^. Mice were initially trained on the rotarod (RotaMex-5, Columbus Instruments, Columbus, OH) for three days: three trials/day at a fixed speed of 5 rpm for 120 s. Mice that fell off the rod were placed back on for the duration of each trial. All animals were allowed a resting period between training trials. The final day of training was completed 3 days prior to the first testing day. Mice were tested on the accelerating rotarod at 10 weeks of age (4–40 rpm over 360 s) three trials per testing day with a resting period between each trial. Total amount of time spent on the rod was recorded by Rotamex system software (Columbus Instruments, Columbus, OH).

### NSC isolation, transduction and formulation

A human neural stem cell line, HK532, isolated from a fetal cortex and conditionally immortalized (US Patent No. 7544511) was further genetically modified by exposing the cells in culture to replication-incompetent recombinant lentivirus expressing EGFP under the human ubiquitin C promoter. The resulting cells were washed free of the transducing virus, further propagated for several passages, and stored frozen to create working stocks. This cell line had been used previously to over-express another potentially therapeutic protein, human IGF-1, using similar method^19^. One day prior to the transplantation surgery, the frozen cells were thawed, washed several times, concentrated to the target cell density of 50 k cells/µL in a proprietary cell suspension medium, and transported to the surgery site overnight by a commercial carrier. Once received, the cell viability was checked by trypan blue exclusion method and found to be 88% viable. The cells were stored on ice until the time of injection and used without further manipulation^20^.

### NSC transplantation

All surgical procedures were conducted in compliance with the UC Davis IACUC policy on rodent survival surgery. Prior to surgery, hair was removed from the head using Nair. Animals were anesthetized with isoflurane (2–3% in oxygen) and placed into the stereotaxic frame. Skin was cleaned with betadine and wiped clean with an alcohol pad. A small incision was made in the scalp to allow visualization of the skull. Two small (1 mm) burr holes were made in the skull at the points of injection. Cells were injected bilaterally into the corpus callosum at − 1.8 mm AP, ± 0.6 mm ML, − 1.8 mm DV relative to bregma using a Hamilton syringe and automated infusion pump (Harvard Apparatus 11 Elite Nanomite) at a rate of 1 µl/min for a total of 4 uL/hemisphere. The needle was withdrawn 0.2 mm after each 1ul injected (Z = − 1.8, − 1.6, − 1.4 and − 1.2). After waiting an additional 1 min after injection, the needle was retracted and the incision sutured with 6–0 silk. A total of 4 × 10^5^ cells were injected per animal (2 × 10^5^ per hemisphere).

Carprofen (5 mg/kg) was administered by subcutaneous injection at the time of surgery and again the following day. All postoperative animals were observed daily for 7 days or until sutures were removed in order to monitor recovery and incision healing.

Mice were euthanized by CO_2_ asphyxiation followed by bilateral thoracotomy 8 weeks post cell implantation (n = 2). Mice were perfused with 10 mL PBS (HyClone) followed by 10 mL formalin (Fisher Healthcare). Brains were harvested, fixed in formalin at room temperature for 24 h, then transferred to 30% sucrose for 24–48 h at 4 °C. Brains were then submerged in dry ice-cooled isopropanol for 5 min, wrapped in foil and stored at − 80 °C until further processing. Brains were prewarmed to − 20 °C and cryosectioned at 30 µM into PSB in a well plate. Sections were then mounted sequentially onto positively charged glass slides and covered with a coverslip using Vectashield antifade mounting media with DAPI (Vector Laboratories). Whole brain sections were scanned, imaged at 20× and stitched together using a Zeiss AxioScan in order to evaluate cell distribution. Additional images were captured using a Keyence BZ-9000 all-in-one fluorescence microscope.

### Statistical analysis

Statistical analysis was performed using GraphPad Prism or Microsoft Excel. One-way ANOVA was used followed by post-hoc Tukey test using GraphPad Prism when appropriate. An unpaired t-test was performed using Microsoft Excel or GraphPad Prism for statistics comparing two groups.

## Discussion

Here we describe a novel immune deficient mouse model for MPSIIIA. This model was created by crossing the immune competent MPSIIIA model with an immune deficient mouse model resulting in a strain that maintains the MPSIIIA phenotype, but lacks T cells, B cell and NK cells. This permits testing of human stem cell therapies for the treatment of MPSIIIA in a relevant animal model.

Animal models play a critical role in the understanding of human disease and the development of therapies. There are currently both spontaneously occurring and genetically engineered models of MPSIIIA. The reduction or absence of SGSH enzyme activity along with the histological lesions of MPSIIIA are the defining features used to identify these models. SGSH activity is undetectable in our novel MPSIIIA-TKO SGSH−/− mice. Histological findings in these MPSIIIA-TKO SGSH−/− mice are consistent with accumulation of storage product in neurons, epithelia, and cells with histiocytic or fibroblastic morphology. These findings are consistent with the original publication by Bhaumik et al. describing the reduced SGSH activity and histologic findings in the immune competent MPSIIIA mouse model^6^.

Two naturally occurring dog models have been described which recapitulate some of the features of the human disease^21,22^. A single New Zealand Huntaway dog, presenting with progressive ataxia, was diagnosed with MPSIIIA based on histological lesions consistent with MPSIII and undetectable levels of SGSH^21^. Additionally, a pair of Dachshund dogs were diagnosed with MPSIIIA after initially presenting with hind limb ataxia. SGSH activity in these dogs was decreased by over 98% and cytoplasmic vacuolation was noted on histology^22^.

Targeted CRISPR/Cas9 mutagenesis was used to create a zebrafish model of MPSIIIA which allows for high-throughput screening of potential therapeutics. Similar to the dogs and mice, this model lacks SGSH activity and exhibits intracellular vacuoles on histology^23^.

The animal models for MPSIIIA all recapitulate the biochemical and histological features of the human disease, although many other hallmarks of MPSIIIA are inconsistent or undetectable in animal models. Behavioral deficits including impaired memory and spatial learning have been reported in the mouse model^9^ and complex behavioral phenotypes are described in the zebrafish model^23^; however, dogs diagnosed with MPSIIIA did not show signs of dementia or display behavioral changes^21,22^. While children with MPSIIIA show a progressive loss of motor skills and progressive ataxia is reported in dogs, debilitating motor deficits are not present in the mouse or zebrafish models. A motor deficit was demonstrated in the present study using the accelerating rotarod at 10 weeks of age where MPSIIIA-TKO SGSH−/− mice spent significantly less time on the rod than their MPSIIIA-TKO SGSH+/+ littermates. While this is not a well-established phenotype in MPSIIIA mice, rotarod performance has been reported as an outcome measure in immune competent MPSIIIA mice^24^; however, the study only compared treatment groups without comparing them to WT controls.

Additionally, some symptoms found in animal models are not present in MPSIIIA patients. MPSIIIA-TKO SGSH−/− mice were found to have severe bladder distension. These findings are consistent with Bhaumik et al. describing the immune competent MPSIIIA mouse model^6^ as well as findings in the MPSIIIB mouse model^25^, yet this is not a feature of MPSIIIA or MPSIIIB in humans^25^.

Hemoglobin, hematocrit, and red blood cell counts were significantly decreased in MPSIIIA-TKO SGSH−/− mice and were, on average, below the reference range. This was an unexpected finding and does not correlate with hematological findings in MPSIIIA patients^26,27^. No evidence of anemia was found in a retrospective study including sixty-one MPS patients where the hemoglobin and hematocrit levels were within normal limits for patients with all types of MPS^26^. Interestingly, bone marrow involvement resulting in anemia is found in several other lysosomal storage diseases including Gaucher disease, Niemann-Pick, Cystinosis, Cholesteryl ester storage disease, Mucolipidosis IV and Aspartylglucosaminuria^27^.

Immune deficient animal models have long been used in immunology, oncology and infectious disease research. Animal models of genetic disease, however, generally have a fully functional immune system, which presents a major barrier for the development of human cellular therapies. One approach to bypass this obstacle is the use of immunosuppressive drugs in traditional disease models. McGinley et al. used oral immune suppression of an Alzheimer’s disease model to characterize and evaluate the efficacy of human cortical neural stem cells engineered to express insulin-like growth factor^19^. Our group used immune suppression delivered by subcutaneously implanted Alzet osmotic pumps^28^ prior to creating immune deficient mouse models to test cellular therapies for Huntington’s disease^16^. Similarly, immune deficient models of Angelman syndrome^29^ and Sandhoff disease have been created to test the efficacy of gene modified human hematopoietic stem cells in relevant disease models^30^. All of these models lack a functional immune system and the ability to reject xenotransplants.

Our MPSIIIA-TKO mice lack helper T cells, cytotoxic T cells, B cells and NK cells as demonstrated by flow cytometry comparing our model to the parental strains. This is consistent with our hematology results showing white blood cells, specifically lymphocytes, well below the normal reference range. Additionally, histologic findings consistent with an immune deficient phenotype (thymic lymphocyte depletion or absence of thymic tissue) were identified in all MPSIIIA-TKO mice evaluated.

Finally, the ability of this novel strain to tolerate xenotransplantation is demonstrated by the robust cell survival and migration of human NSCs in the brain 8 weeks post cell transplantation in MPSIIIA-TKO mice. Our current work lays the foundation for future studies using MPSIIIA-TKO mice to test novel stem cell therapies aimed to treat MPSIIIA.

## Conclusion

This novel, immune deficient MPSIIIA mouse model has the potential to be extremely useful for testing human cell therapies for the treatment of MPSIIIA as it retains the disease phenotype but eliminates the ability of the mice to reject human cells.

### Supplementary Information

Supplementary Information.

## Data Availability

The data that support the findings of this study are available from the corresponding author upon reasonable request.
