# Supplementary material for: An immune deficient mouse model for mucopolysaccharidosis IIIA (Sanfilippo syndrome)
Source: Sci Rep. 2023 Oct 27;13:18439. doi: 10.1038/s41598-023-45178-0 (PMC10611714; doi:10.1038/s41598-023-45178-0)
Supplement: Supplementary file 1 — Supplementary Information. [file 41598_2023_45178_MOESM1_ESM.docx]

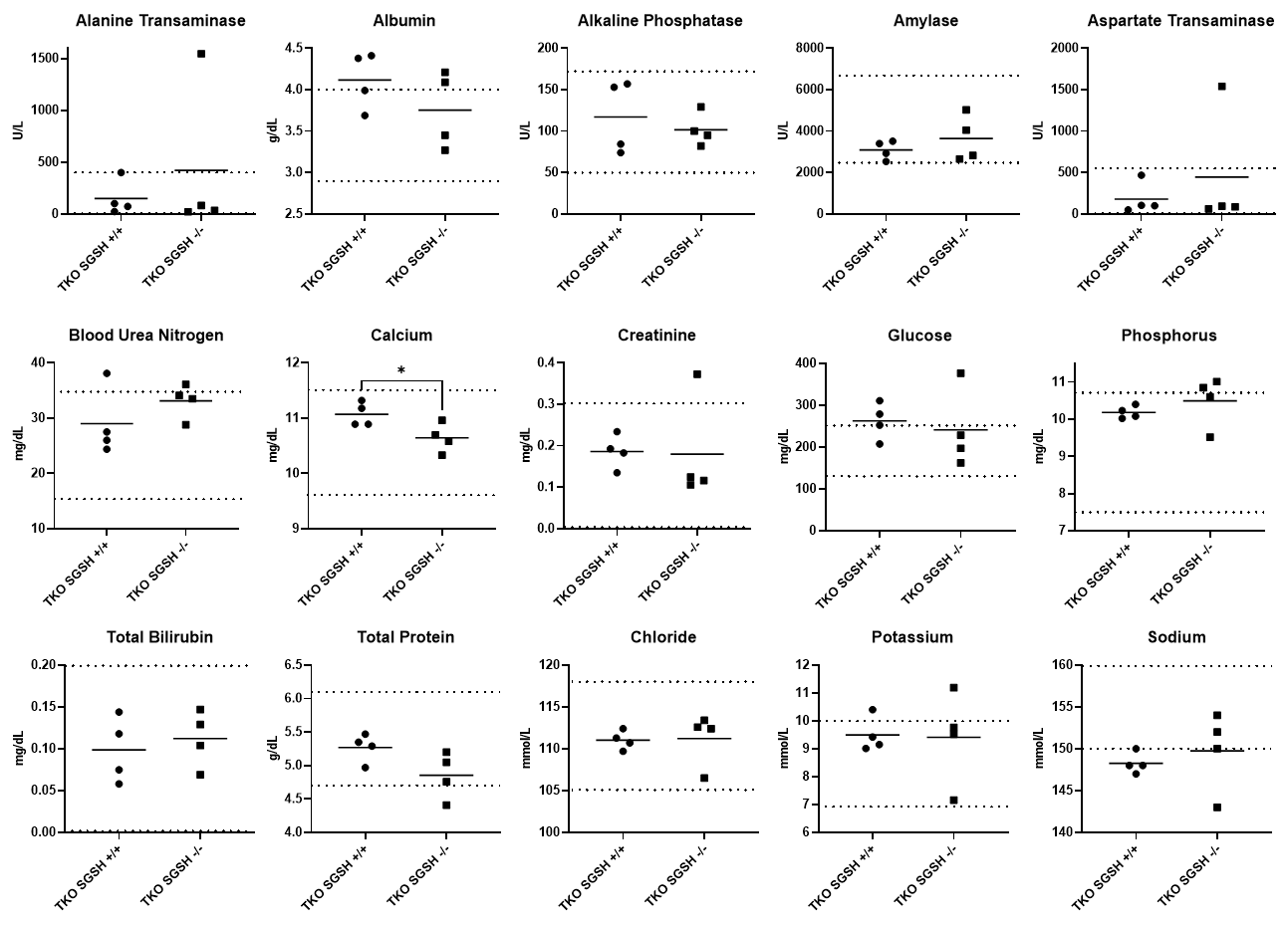


**Supplementary Figure 1:** Hematological findings in 8-month-old MPSIIIA TKO mice. Dotted lines define the reference range. * *p* < 0.05


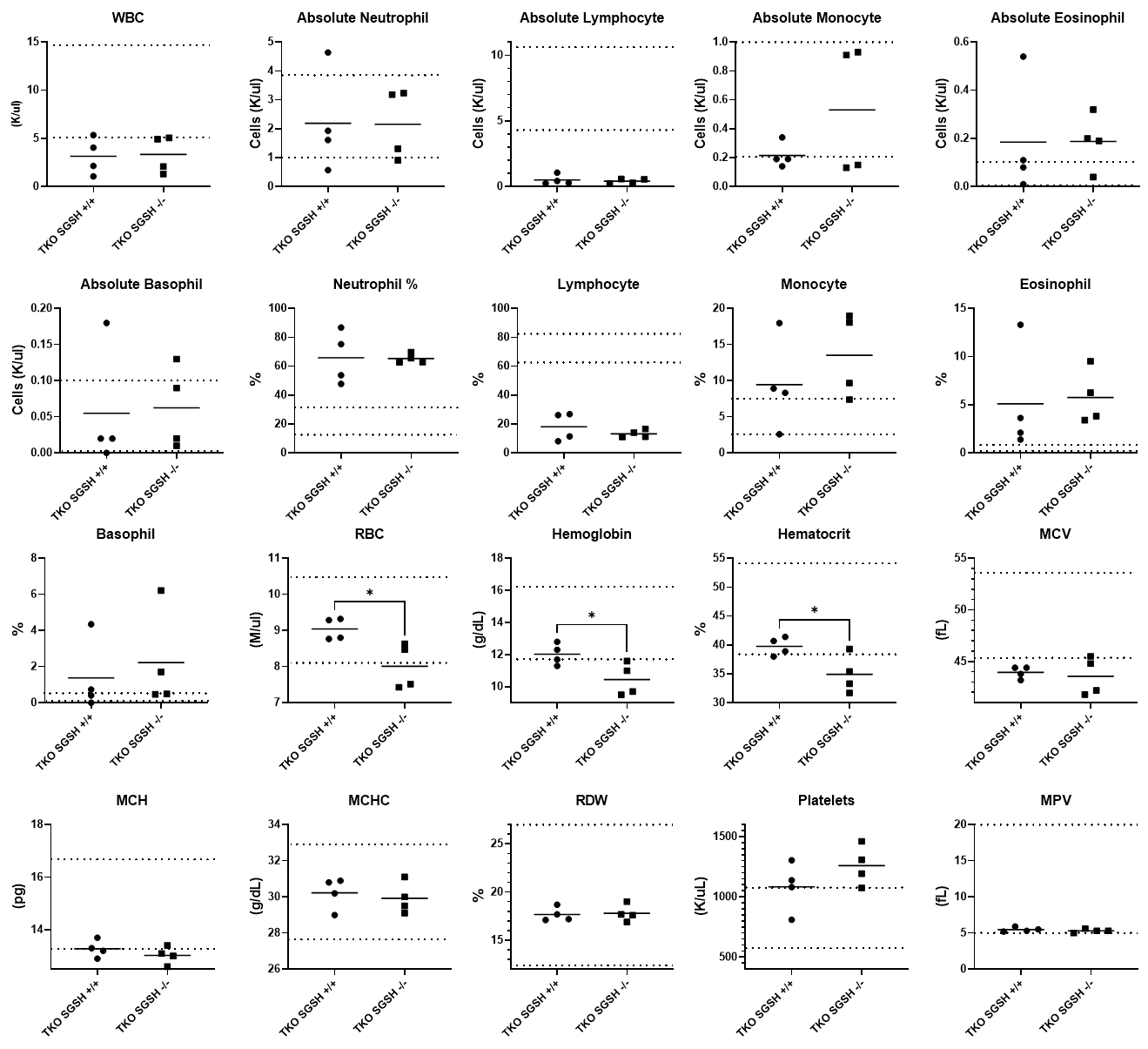


**Supplementary Figure 2:** Hematological findings in 8-month-old MPSIIIA TKO mice. Dotted lines define the reference range. * *p* < 0.05

| **Tissue** | **Cell type/s affected** | **Storage product** | **Distribution** |
| --- | --- | --- | --- |
| Pituitary gland | Histiocytic/fibroblastic | 2 | Multifocal |
| Thyroid | Epithelial | 2 | Multifocal |
| Parathyroid gland | Epithelial | 2 | Multifocal |
| Pancreas | Epithelial, histiocytic/fibroblastic | 1-2 | Multifocal |
| Salivary gland, acini, ducts | Epithelial | 2 | Multifocal |
| Liver | Kupffer cells, hepatocytes | 2-4 | Multifocal |
| Gallbladder | Epithelial, histiocytic/fibroblastic | 2 | Multifocal to diffuse |
| Kidney | Epithelial, histiocytic/fibroblastic | 2-3 | Multifocal |
| Urinary bladder, ureter, urothelium | Epithelial, histiocytic/fibroblastic | 2 | Multifocal |
| Brain, leptomeninges | Histiocytic/fibroblastic | 1-2 | Multifocal |
| Adrenals | Histiocytic/fibroblastic | 2 | Multifocal |
| Heart | Cardiomyocytes | 1-2 | Multifocal |
| Diaphragm, perimysium | Histiocytic/fibroblastic | 2 | Multifocal |
| Tongue, interstitium | Histiocytic/fibroblastic | 2 | Multifocal |
| Spleen, red pulp | Histiocytic/fibroblastic | 2 | Multifocal |
| Gastrointestinal tract | Histiocytic/fibroblastic | 1-2 | Multifocal |
| Skin | Histiocytic/fibroblastic | 2-3 | Multifocal |
| Vagina | Histiocytic/fibroblastic | 2-3 | Multifocal |
| Uterus | Epithelial, histiocytic/fibroblastic | 2-3 | Multifocal |
| Ovary | Histiocytic/fibroblastic | 3 | Multifocal |
| Oviduct | Histiocytic/fibroblastic | 2 | Multifocal |
| Preputial/clitoral gland | Histiocytic/fibroblastic | 2 | Multifocal |
| Mammary gland | Epithelial, histiocytic/fibroblastic | 2 | Multifocal |
| Testes | Epithelial, histiocytic/fibroblastic | 2 | Multifocal |
| Epididymis | Histiocytic/fibroblastic | 2 | Multifocal |
| Penis, corpus cavernosum glandis | Histiocytic/fibroblastic | 2 | Multifocal |
| Seminal vesicles, prostate | Epithelial, histiocytic/fibroblastic | 2 | Multifocal |
| Lung | Vascular smooth muscle | 1-2 | Multifocal |

**Supplementary Table 1:** Grading reflects the relative number of cells with storage product; 1=mild involvement, 2=moderate involvement, 3=severe involvement. Abbreviations: DCT=distal convoluted tubule, CD=collecting ducts
